# Liver transplantation in Acute-on-Chronic liver failure: Timing of transplantation and selection of patient population

**DOI:** 10.3389/fmed.2022.1030336

**Published:** 2022-12-08

**Authors:** Xue Li, Liang Zhang, Chunmei Pu, Shanhong Tang

**Affiliations:** ^1^Department of Gastroenterology, The General Hospital of Western Theater Command, Chengdu, China; ^2^School of Medical and Life Sciences, Chengdu University of Traditional Chinese Medicine, Chengdu, China

**Keywords:** Acute-on-Chronic liver failure, liver transplantation, prognosis, APASL, EASL

## Abstract

Acute-on-Chronic liver failure (ACLF) is a clinical syndrome with high short-term mortality. Alcoholic ACLF is prevalent in European and American countries, while hepatitis B virus (HBV)-related ACLF is more common in the Asia-Pacific region. There is still a lack of a unified definition standard for ACLF, due to various etiologies and pathogeneses in different continents. Currently, liver transplantation (LT) is the most effective treatment for liver failure. However, the shortage of liver sources is still a global problem, which seriously limits the clinical application of an LT. Premature LT aggravates the shortage of liver resources to a certain extent, and too much delay significantly increases the risk of complications and death. Therefore, this study reviews the current literature on LT in the treatment of ACLF and discusses further the challenges for ACLF patients, the timing of LT for ACLF, and the choice of the patient population.

## Introduction

Acute-on-Chronic liver failure is characterized by an extreme fatigue, a rapid deepening of jaundice, coagulation disorder, and decompensated ascites, with or without hepatic encephalopathy. It progresses rapidly with a poor prognosis and the short-term (28-day) mortality can reach as much as 23–74% (1, 2). Acute viral hepatitis, virus reactivation, bacterial infection, heavy drinking, and drug/toxic injury are common inducements of ACLF, but about 40% of events have no clear inducement (3). There is still a lack of a unified definition standard for ACLF, due to various etiologies and pathogeneses in different continents. Currently, the main treatment method for liver failure is LT, an artificial liver support system (ALSS), and standard medical treatment (SMT). But there is no specific drug for a comprehensive medical treatment, and artificial liver is effective only for some patients. For patients with poor treatment effects of internal medicine and artificial liver, the most effective and fundamental treatment method is LT (4), which increases the 6-month survival of patients with ACLF grade 2 and grade 3 from 10 to 80% (5). The shortage of liver sources, the high cost of LT, and the immunological rejection limit the clinical application of a liver transplantation (LT). So far, there has still been a lack of a clear risk prediction model and appropriate guidelines for the LT treatment of ACLF to judge which patients can benefit from the LT. The timing of treatment fixed for an LT has also aroused controversy. Therefore, it is significant to further explore which segment of the patient population can obtain a better prognosis from LT.

## Etiology and diagnosis of ACLF

### Etiology

Artificial liver support system is the most common type of liver failure. In Eastern countries, hepatitis B virus (HBV) reactivation and alcoholic hepatitis are common predisposing factors for ACLF, while in Western countries, infection and alcoholic hepatitis are the main causes of ACLF (6). In recent years, alcoholic hepatitis has also become one of the commonest causes of ACLF in several regions of Asia. ACLF is a special pathophysiological state that involves a liver injury. The injury triggers the disorder and an extensive activation of the inflammatory cytokine pathway, which leads to a systemic inflammatory response syndrome (SIRS), single organ dysfunction, and which finally develops into multiple organ dysfunction syndromes (7). The systemic inflammatory response is the main feature of the ACLF, leading to a significant increase in short-term mortality. While cirrhosis decompensation, the occurrence of new infections, the severity of inflammation, and multiorgan failure (MOF) are all associated with high mortality. MOF with or without sepsis accounted for 90% of deaths (8). In accordance with the findings of Petrowsky et al. (9) among the risk factors, they showed that infection was associated with a higher risk of futile transplantation (90-day mortality). Bacterial infections and the subsequent development of organ failure are the main determinants of mortality in ACLF patients on the waiting list, and thus, it is important to recognize early the subgroup of patients who are at high risk of developing such complications. According to the previous research (10), the 28- and 90-day non-transplant mortality rates of ACLF patients were 32 and 49.8%, respectively, significantly higher than those of patients with chronic liver disease. The 28-day non-transplant survival rate of ACLF in Europe and America is about 70% (11). In China, the 28-day non-transplant survival rate of ACLF is only 50% (12, 13). Without LT, the prognosis of ACLF patients is very poor. To sum up, ACLF progresses rapidly. Excessive inflammatory response and immune imbalance are the core pathogenesis of ACLF. The occurrence of acute events, such as pro-inflammatory events, viral reactivation, and acute viral hepatitis, aggravates liver injury, thus leading to more complications and poorer prognosis. Table 1 summarizes the non-transplantation-related outcomes in part of studies of the ACLF.

**Table 1:** Non-transplantation-related outcomes in part of studies of Acute-on-Chronic liver failure (ACLF).

**References**	**Criterion**	**ACLF grade**	**Non-LT survival/mortality**
Moreau et al. (14)	EASL	CLIF 1-3	The 90-day mortality rate increased from 40.7 to 52.3 to 79.1%
Choudhury et al. (15)	APASL	AARC I, II, III	The 90-day mortality rate is 12.7, 44.5, 85.9%
Artru et al. (16)	EASL	MELD ≥ 35	The 1-year survival rate is 7.9%
Gustot et al. (17)	EASL	d3–7 CLIF 3	The 28-day mortality rate is 100%
Zhang et al. (18)	COSSH	ACLF 2–3	The 28-day survival rate is 6.8%

### Diagnosis and classification

The definitions proposed by the European Association for the Study of the Liver (EASL) (19) and the Asian Pacific Association for the Study of the Liver (APASL) (20) are widely used. The definition of ACLF in Europe and America emphasizes the susceptibility and multiple organ failures caused by liver cirrhosis. The diagnosis and classification of ACLF and the determination of transplant candidates in European and American countries mainly focus on acutely decompensated cirrhosis, multiple organ failures, and 28-day high mortality. According to the condition of ACLF patients with organ failure, EASL classifies the ACLF into three categories: ACLF 1: single organ kidney failure; or single failure of the liver, coagulation, respiration, and elevated creatinine of 1.5–1.9 mg/dl or hepatic encephalopathy (HE) (14) grade I or II; or HE grade III/IV and elevated creatinine of 1.5–1.9 mg/dl; ACLF 2: 2 organ failures; ACLF 3: ≥3 organ failures. Studies (21) have also validated EASL-CLIF grading in patients with cirrhosis. The 90-day mortality rate associated with ACLF 1–3 non-LT increased from 40.7 to 52.3 to 79.1%. The APASL proposed the diagnostic criteria for ACLF based on the expert consensus (5): “In patients with or without previously diagnosed chronic liver disease, acute liver injury is followed by jaundice [total bilirubin (TBil) >50 mg/L (>85.5 μmol/L)] and coagulopathy [international normalized ratio (INR) >1.5 or prothrombin activity <40%], with ascites and/or hepatic encephalopathy within 4 weeks of onset.” The definition of ACLF in APASL focuses on liver failure. The current ACLF definition was reassessed based on the AARC database. According to the AARC score, ACLF was divided into three grades: Grade I was 5–7, grade II was 8–10, and grade III was 11–15. The 28-day mortality rates were 12.7, 44.5, and 85.9%, respectively (15).

## Prognostic model of ACLF

So far, multiple prognostic scores have been used, including Child–Turcotte–Pugh (CTP) score, the Model for end-stage liver disease (MELD) score, the MELD sodium (MELD-Na) score, APASL ACLF Research Consortium (AARC) score, chronic liver failure-sequential organ failure assessment (CLIF-SOFA) score, etc. The CTP model was first proposed in 1964, which is a commonly used classification standard for quantitative assessment of liver function in patients with liver cirrhosis. The CTP score is a classical parameter that is widely used to evaluate the liver reserve function and assess the condition and prognosis of patients with liver cirrhosis. However, a major drawback with the CTP score is that it is subjective, especially for ascites and hepatic encephalopathy, which makes it difficult to convert to objective grading. And due to the narrow grading window, sometimes it cannot accurately reflect the severity of the patient's condition. The MELD score is used to predict the short-term mortality of patients with chronic liver disease and awaiting transplantation. Moreover, it also serves as the main tool for liver source allocation in Eastern and Western countries. However, the MELD score refers only to creatinine, bilirubin, prothrombin time, and INR, which has certain limitations in the practical clinical application of LT. Among MELD-derived models, the MELD-Na scoring model has been supported by a large number of studies. Compared with creatinine, serum sodium can predict renal function injury earlier and in a more sensitive manner. In a prospective study by Biggins et al. (22), the risk of death in patients awaiting transplantation was found to be linearly related to sodium levels. In the studies of Machicao et al. (23) and Sharma et al. (24), it was also further proved that MELD-Na model had more advantages and value than MELD in predicting the mortality rate after transplantation waiting list or LT. The MELD score and the MELD-Na have been implemented as objective prognostic indicators to decrease mortality rates of those patients on the waiting list (25). CLIF-SOFA is an important assessment system for organ damage in ACLF, which can be used to select treatment options including LT (26). Jalan et al. (27) proposed a simplified EASL-CLIF score based on CLIF-SOFA, based on the number of organ failures, and reported that it had a larger area under the receiver operating characteristic curve (ROC) than the MELD-Na score in predicting the 90-day mortality in ACLF. In the EASL recommendations, it was suggested that patients with acute decompensation of liver cirrhosis should consider using the CLIF-SOFA scoring system. Recent studies (28) show that the CLIF-ACLF score improved the 28- and 90-day mortality predictions by about 25–28%, as compared to the CTP, MELD, and MELD-Na scores (29). The AARC-ACLF score was established based on the end-stage liver disease model (MELD) and lactate. The indicators are easier to obtain in clinical work, and the calculation process is simple and convenient. Choudhury et al. (15) included 1,021 ACLF cases that met the definition of APASL guidelines. After 90 days of follow-up, it was found that these ACAL cases were sensitive to early mortality, but the specificity was poor. The AARC-ACLF was established to predict short-term mortality and LT needs in ACLF patients, and it has been demonstrated to have a better prognostic value than CLIF-SOFA (20). In 2018, the Chinese Severe Hepatitis B Study Group (COSSH) released the Chinese criteria for the diagnosis and grading of HBV-ACLF and the COSSH-ACLF score (13). Compared with MELD score, COSSH-ACLF score increases with age, HE grades, circulation and respiration indicators, and compared with AARC score, COSSH-ACLF score increases with age, circulation and respiration indicators, and makes up for the shortcomings of the EASL-ACLF standard that is not suitable for HBV-infected population, so that it can better reflect the prognosis of HBV-ACLF patients. Table 2 describes the advantages and limitations of the various prognostic models for ACLF.

**Table 2:** The advantages and limitations of the various prognostic models for Acute-on-Chronic liver failure (ACLF).

**Model**	**Parameters**	**Advantages**	**Limitations**
CTP	HE; Ascites; Alb; PT; INR	The calculation is simple, the evaluation index is easy to obtain clinically, and the impact of portal hypertension on the disease is taken into account	HE and ascites grading have certain subjectivity in clinical practice, and sometimes they cannot truly reflect the severity of the patient's condition and lack effective indicators to evaluate renal function
MELD	TBil; Cre; INR; Causes	The parameters are objective, taking into account renal failure, liver failure and coagulation disorders, and can accurately evaluate the condition of patients with liver failure	The failure of organs such as brain and circulation, portal hypertension, ascites and other complications were not considered, resulting in the inconsistency between the severity of the patient's condition and the MELD level, and it is easy to miss the best transplantation window period
MELD-Na	TBil; Cre; INR; Causes; Na	Compared with Cre, serum sodium can predict renal function damage earlier and more sensitive, which is mainly suitable for patients with hyponatremia	The impact of extrahepatic organ failure on the prognosis of patients is not fully taken into account, which cannot truly reflect the severity of the underlying systemic disease. In addition, the serum sodium level is easily affected by many factors such as blood volume and diuretics
EASL-CLIF	HE; TBil; Cre; INR; MAP; PaO2/FiO2; SPO2/FiO2; WBC	The severity of liver, renal, central nervous system, coagulation, circulatory, and respiratory function was comprehensively assessed to further stratifiate patients in different mortality risk subgroups	It is established based on Westerners who mostly have alcohol-related liver disease and hepatitis C, and the ability to evaluate HBV-ACLF needs to be explored, and the calculation of the scoring model is complex, which is not good for clinical operation
AARC	HE; TBil, INR, Cre; lactate	The calculation is simple, the index is easy to obtain, and covers the functional evaluation of other systems, which is more suitable for the actual situation of ALCF patients in the Asia-Pacific region	At present, there are few studies on the application of this model, and prospective and multi-center validation is needed
COSSH	HE; TBil; INR; Neut; Age: BUN	The model was established based on a multi-center and large sample of hepatitis B patients, and the risk stratification of HBV-ACLF was optimized	The population is HBV-ACLF patients, and the prognosis of ACLF caused by other causes is not considered. However, too many scoring indicators are not good for clinicians to quickly evaluate the prognosis and disease classification of ACLF patients

In conclusion, MELD, CLIF-SOFA, and AARC scores have been recorded as suitable for predicting mortality in patients with ACLF. However, due to inconsistent definitions of ACLF and various etiologies between the East and the West, these models do not have a stable and perfect predictive power. Whether these models can accurately reflect the clinical severity of ACLF patients requires a further study, and The American Association for the Study of Liver Diseases (AASLD) also advises against relying solely on the currently available prognostic scoring systems to predict outcomes and identify candidates for LT. Therefore, there is still a lack of a clear risk prediction system for judging the timing of liver transplantation in patients with an advanced liver failure. If the timing is too early, the liver source will be wasted, and if it is too late, the timing of the operation will be lost or the prognosis will be poor. As the course of ACLF changes rapidly and is associated with high short-term mortality, it is important to identify patients for LT before the onset of the development of MOF. At present, there is no optimal allocation system for liver transplantation, and the choice of SMT, LT, or palliative treatment is still an urgent problem to be solved. More prospective studies are needed to formulate the best prognostic criteria in the future.

## Current status of non-transplant therapy

The latest research based on the CANONIC database showed that most of the patients with d3–7 ACLF 2 or 3 died in the first month of follow-up (death rates were 57 and 87%, respectively) (19). At present, the non-transplantation treatment for ACLF patients mainly deals with the acute injury factors that cause ACLF. The management of patients with liver failure aims to maintain or restore vital organ functions and prevent the development of MOF. However, the lack of liver sources, the high cost of transplantation, and immunological rejection greatly limit the development of liver transplantation (LT). In this case, artificial liver support system (ALSS) is very suitable to buy time for spontaneous liver regeneration or emergency liver transplantation, and becomes an important bridging therapy (30). Due to different patient conditions and support modes, the efficiency reported is also different, about 50–80%. Currently, non-biological artificial liver (NBAL) is widely used, mainly including plasma exchange (PE), double plasma molecular absorption system (DPMAS), molecular adsorbent recycling system (MARS), etc. PE has been partially studied in HBV-related ACLF. Some non-randomized trials have shown that compared with standard treatment, HBV-ACLF patients treated with PE have benefit in survival. However, at present, PE evaluation is not being conducted for ACLF patients from other causes (31). Studies Fernández and Saliba (32) have shown that the survival rate of ACLF patients of any grade after treatment with ALSS is higher than that of patients without such treatment. In 1993, Stange et al. (33) established MARS, which can improve the renal dysfunction and the 30-day survival rate. In a multicenter clinical trial involving 180 patients with ACLF, it was found that MARS had no significant difference in the survival rate of patients compared with standardized treatment. The 28-day mortality rates of patients treated with MARS and conventional treatment were 41 and 40%, respectively (34). Gerth et al. (35) arrived at the same conclusion in a study of 101 patients with Acute-on-Chronic liver failure grade 3 (ACLF 3). Compared with other ALSS treatment groups, the MARS treatment group can significantly reduce the 14-day mortality (6.4 vs. 27.8%), but it has no significant effect on mortality within 21-days. Larsen et al. (36) have shown that PE and MARS treatment can stabilize patients' hemodynamics and improve blood biochemical indicators, but PE can ultimately improve the survival rate of patients with ACLF. The underlying reason may be related to the fact that PE can not only remove toxins but also supplement coagulation factors and other beneficial substances lacking in patients with liver failure. The 2019 APASL guidelines clearly recommend PE as one of the effective bridging therapy options for ACLF patients before LT. In a prospective study conducted by Maiwall et al. (37), it was found that PE could improve systemic inflammation and reduce the occurrence of MOF in patients with ACLF, and that the PE treatment reduced liver failure-related death at 90-day, which may be the preferred mode of ALSS in ACLF.

The ALSS treatment combined with LT in patients with HBV–ACLF improved short-term survival. ALSS treatment pre-LT is an independent protective factor affecting the 4-week survival rate after LT (38). Ling et al. (39) found that decreasing MELD score (to <30) in ACLF using an ALSS as bridging therapy improved outcomes in the responders to levels similar to those who had upfront LT. In patients who did not improve their MELD score to <30, the survival was poor, despite transplantation. Considering the different diagnostic criteria for ACLF, the different disease severities of the included patients, and the differences in the mode and frequency of ALSS treatment, no consensus had been reached.

In 2019, the APASL updated the definition of ACLF, pointing out that one of the key characteristics of ACLF is reversible. Over time, liver injury, fibrosis, and portal pressure have been gradually reduced and liver reserve improved in some patients. It emphasizes that early death risk judgment and clinical intervention can improve the prognosis of patients. This is also the golden period for bridging therapy before transplantation and for promoting the ability of liver regeneration and repair.

## Prognosis related to liver transplantation

### Transplant timing assessment

Liver transplantation includes living donor liver transplantation (LDLT) and deceased-donor liver transplantation (DDLT). Some studies (40) have found that vascular and biliary tract-related complications are the main challenges faced by the LDLT recipients, whereas kidney injury and recurrent cirrhosis are the main problems after DDLT. In the allocation of liver sources, the principle of “optimal condition” is followed. The evaluation system for ACLF transplantation mainly relies on the MELD score, and both DDLT and LDLT can be used for ACLF patients. However, the serious shortage of deceased-donor liver resources, especially in Eastern countries, makes LDLT an important surgical option to save the lives of ACLF patients. At present, patients with MELD scores ≤18 make up a large proportion of transplant waiting lists worldwide, but studies have shown that MELD scores <14 do not show a significant survival benefit (41). Patients with MELD ≥15 are recommended to be placed on the waiting list for the 2013 practice guidelines by the American Association for the Study of Liver Diseases (AASLD) and the American Society of Transplantation 2015 (42). Moreau et al. (14) recruited more than 90 patients from various European countries with different etiologies and found a close correlation between the prevalence of ACLF in each country, short-term mortality, and the prevalence of LT. Studies have shown that most ACLF 1 and ACLF 2 patients have a good prognosis after LT, and the 1-year survival rates are 77–92 and 72–88%, respectively (16). Gustot et al. (17) found that among grade 3 patients, the survival was 78% after LT. In the study by Biggins et al. (22), the risk of death within 6 months of waiting for a liver transplant was found to be 6, 16, and 37% for MELD-Na scores of 20, 30, and 40, respectively. Huebener et al. (43) evaluated the risk of LT in patients with ACLF. Compared with no-ACLF patients, ACLF is associated with significantly higher short-term incidence and mortality after LT (90-day survival rate of non-ACLF patients is 96.1%, while that of ACLF patients is 72.4%, *P* < 0.0001). As regards patients with no improvement at the time of transplantation, the ACLF score, CLIF-ACLF score, CLIF-OF (organ failure) score, and MELD score are significantly lower. Yadav et al. (44) used EASL-CLIF to define ACLF and found that the 1-year post-transplant survival rate of patients with ACLF was 85%. A study that used the APASL-ACLF definition reported a 1-year survival rate of 87% (20). Different from European and American countries that ACLF entered the transplant waiting list earlier, in the Asia-Pacific region and other countries for a variety of reasons, LT is generally not recommended for patients until serious complications set in and multiorgan failures (MOFs) occur. The Chinese Society of Hepatology recommended in the 2019 Edition of Guidelines for The Diagnosis and Treatment of Cirrhosis that patients with MELD scores ≥12 are included in the waiting list for LT, and patients with MELD scores ≥18 need LT (45). The MELD score ≥ 35 is closely associated with higher mortality on the waiting list. However, the literature (46) shows that LT can provide acceptable long-term survival results, even in subjects with MELD scores ≥ 40 without pre-transplant sepsis, cardiac risk, or multiple diseases. Zhang et al. (47) used the Chinese Severe Hepatitis B Study Group-ACLF (COSSH-ACLF) score and found that reassessment of the COSSH-ACLF grade at d3–7 days after diagnosis could indicate emergency LT. The 28-day survival rate was 93.3% for baseline ACLF 3 and d3–7 ACLF 2 or 3 who received LT, and 6.8% for patients who did not receive transplantation (*P* < 0.0001).

LT may be the basic treatment for ACLF to reverse the extrahepatic MOF. The median transplant-free survival in patients with ACLF was 48-day, and previous guidelines have mentioned that an LT was recommended, if the expected 5-year survival exceeded 50% (48). Patients who are able to receive a liver transplant generally have better outcomes, especially those who have improved under conservative treatment measures.

### Timing of LT for patients with MOF and high grade

As ACLF is a heterogeneous condition and follows a dynamic course, the decision to undergo LT should be individualized. The survival rate after liver transplantation is good, and the 5-year survival rate is 74–90% (49). However, the pre-transplant condition of ACLF patients is usually critical and often complicated by the high incidence of infection and MOF. About half of the patients died while waiting for LT. As described in the CANONIC study, the ACLF 28-day mortality was 33%, with mortality observed to increase to 80% with three or more failing organ systems. Transplantation presents the best outcomes in patients with ACLF who do not recuperate spontaneously or who do not ameliorate with supportive treatments (50). A retrospective study (51) in Spain showed that patients with ACLF 3 had a survival rate of 83.9% after transplantation and only 7.9% of patients with ACLF 3 had a survival rate without LT. Moon et al. (52) retrospectively analyzed the data of patients with liver cirrhosis who underwent LDLT and found that the 5-year survival rate and graft survival rate of patients with a high MELD score (≥30) were 76.4 and 75.2%, respectively. The 1-, 3-, and 5-year survival rates of grafts and recipients in the ACLF group were 76.8, 72.1, 70.5 and 79.5, 73.6, 72.1%, respectively. Through timely LDLT treatment and comprehensive perioperative management, ACLF patients can obtain an ideal long-term survival after operation. In patients with high MELD scores awaiting transplantation, the optimal timing of LDLT should be scheduled before the progression to ACLF. Yadav et al. (44) also found that LDLT had a good survival rate in patients with ACLF. The 1-year survival rates of patients with ACLF 1, ACLF 2, and ACLF 3 were 92.9, 84.5, and 75.6%, respectively, who received LDLT, and LDLT donors did not face waste problems due to insignificant recipient treatment. As the course of ACLF changes rapidly and higher ACLF grades are associated with high short-term mortality, it is important to identify patients for LT before the development of MOF. Given the high mortality of ACLF 3 without transplantation, LT is a potentially important intervention for these patients. In some reports (53, 54) from large LDLT centers, high MELD scores did not affect graft and patient survival. Although ACLF 3 patients showed good survival after LT, they underwent prolonged hospital stays and exhibited a higher incidence of pulmonary infections and renal complications after transplantation than patients without or with minor organ failure (55). Table 3 summarizes LT-related outcomes in part of studies of ACLF.

**Table 3:** Liver transplantation (LT)-related outcomes in part of studies of Acute-on-Chronic liver failure (ACLF).

**References**	**Criterion**	**ACLF grade**	**LT survival/mortality**
Sarin et al. (20)	APASL	ACLF	The 1-year survival rate is 87%
Ling et al. (39)	APASL	ACLF	The 5-year survival rate is 73%
Artru et al. (16)	EASL	CLIF-ACLF1–2	The 1-year survival rate is 77–92 and 72–88%, respectively
Gustot et al. (17)	EASL	CLIF 3	The 1-year survival rate is 78%
Huebener et al. (43)	EASL	CLIF 3	The 90-day survival rate is 52.4%
Yadav et al. (44)	EASL	CLIF	The 1-year survival rate is 85%
Zhang et al. (18)	COSSH	ACLF2–3	The 28-day survival rate is 93.3%

In conclusion, decisions to undergo LT or not should be individualized. To evaluate ACLF effectively, the required model must be not only dynamic but also comprehensive. Prospective studies are still needed to further evaluate and determine the optimal timing and selection criteria for transplantation in ACLF.

### The “Golden Window” of LT and liver regeneration

As the transplantation window of ACLF patients is very short, the short-term mortality is high, and the occurrence of organ failure is also very important for prognostic prediction (56). The ACLF patients are susceptible to infection and the early graft-free survival rate is very low. Artzner et al. (57) stratified LT in critically ill patients with cirrhosis based on pre-transplantation factors and established TAM (Transplantation for ACLF-3 patients Model) scores, a finding that the model helped stratify post-transplantation survival in ACLF 3 patients and determine the optimal transplantation window. All treatment methods, except LT, rely on the powerful regeneration ability of the liver. At present, there are no clear criteria for screening ACLF patients for LT, and there are no effective prognostic markers for evaluating the recovery of clinical treatment. In recent years, alpha-fetoprotein (AFP) has turned into a prognostic marker of acute liver failure and is used as a serum marker of liver regeneration (58). In the previous work, our center conducted a long-term clinical observation and follow-up on the AFP level of HBV-ACLF patients. It was found that AFP is also an independent predictor of ACLF prognosis, in addition to age, TBil, and INR. It was speculated that AFP can be used as a useful marker to predict the prognosis of HBV-ACLF. A high AFP level often indicates a better prognosis (59). Combined with clinical indicators such as liver regeneration and organ damage, our center established a new scoring system to predict the prognosis scoring system (TACIA score) of HBV-ACLF patients within 3 months, and found that it has a better short-term prognosis for patients to evaluate effectiveness (60).

Monitoring inflammatory markers and regenerative markers during the “Golden Window” of ACLF progression will also help us to judge the prognosis of patients. In future studies, we should pay attention to the impact of the inflammatory response on the survival and prognosis of patients. In addition, the process of ACLF is potentially reversible, so whether liver function is compensated or not is very important for patients with liver failure. An early diagnosis and timely intervention of ACLF alone can protect surviving hepatocytes as much as possible from inflicting further damage to liver cells, and thereby create favorable conditions for liver regeneration.

Generally speaking, the results of ACLF patients after LT are worse than those of chronic liver disease alone. This may be due to the severity of the underlying liver disease, which is manifested as higher MELD, organ dysfunction, and systemic inflammatory response syndrome. Zhang et al. (18) propose early transplantation and creation of a Markov decision process model, as opposed to waiting for an optimal donor organ or improvement in the number of MOFs. Artru et al. (16) found that compared with the non-LT patient group, LT had a significant effect on ACLF 3 patients. Due to the short time of an assumed “transplant window,” they recommend that patients undergo rapid management procedures and that patients with ACLF 3 must be immediately referred to a specific liver intensive care unit. Therefore, an effective treatment during this period alone could help improve an acute injury, regulate the patient's immune response, prevent the development of sepsis, promote liver regeneration, reverse the MOF, and consequently reduce mortality.

## Future directions

Acute-on-Chronic liver failure is a common liver disease syndrome and treatment is mainly based on the prevention of organ failure and its associated complications. LT has a good effect on ACLF 1–2 patients. While for ACLF 3 without LT, the prognosis is poor. With the rapid changes taking place in the ACLF process, it is important to carry out a comprehensive medical treatment as well as anti-infective preventive measures in time, and to enter the LT evaluation process as soon as possible to screen the most suitable LT recipients and determine the golden time for surgery. Based on the existing evidence and prospects, some improvements can be brought about in the ACLF field, which may help to improve the management and prognosis of patients. At present, LT can improve the prognosis of patients with different degrees of ACLF. In the future, it is necessary to establish an LT risk assessment model and screen high-risk groups, so that patients can be managed more scientifically at admission, and the correct intervention and monitoring measures carried out as soon as possible to obtain the best treatment effect, thereby improving the prognosis of patients with ACLF and the effectiveness and efficiency of LT. It is expected that in the future, a large sample and multi-center prospective clinical research results will guide the formulation of more accurate clinical diagnostic criteria and a prognostic scoring system to determine the needs and appropriate time of LT in the patient population. The accumulation of long-term follow-up data will also contribute to the formulation of future clinical guidelines.

## Author contributions

XL performed literature searches and prepared the initial draft. LZ, CP, and ST reviewed and helped to finalize the manuscript. All of the authors read and approved the manuscript.
